# Nutritional Aspects to Cardiovascular Diseases and Type 2 Diabetes Mellitus

**DOI:** 10.1007/s11886-023-02018-x

**Published:** 2024-01-23

**Authors:** Hayley E. Billingsley, Emily M. Heiston, Moriah P. Bellissimo, Carl J. Lavie, Salvatore Carbone

**Affiliations:** 1 Department of Internal Medicine, Division of Cardiovascular Medicine, University of Michigan, Ann Arbor, MI USA; 2 Division of Cardiology, Department of Internal Medicine, Pauley Heart Center, Virginia Commonwealth University, Richmond, VA USA; 3 Department of Cardiovascular Diseases, Ochsner Clinical School, John Ochsner Heart and Vascular Institute, The University of Queensland School of Medicine, New Orleans, LA USA; 4 Department of Kinesiology & Health Sciences, College of Humanities & Sciences, Virginia Commonwealth University, Richmond, VA USA

**Keywords:** Obesity, Weight loss, Type 2 diabetes mellitus, Diet, Nutrition, Mediterranean diet

## Abstract

**Purpose of Review:**

In this narrative review, we discuss the current evidence related to the role of dietary interventions to prevent and treat type 2 diabetes mellitus (T2DM) and cardiovascular disease (CVD). We also propose alternative therapeutic strategies other than weight loss in this population, namely, improvements in cardiorespiratory fitness and its determinants.

**Recent Findings:**

While weight loss has been consistently associated with the prevention of T2DM and improvements in glycemic control in those with established diseases, its role in preventing and treating CVD is less clear. In fact, in this setting, improvements in diet quality have provided greater benefits, suggesting that this might represent an alternative, or an even more effective strategy than energy-restriction.

**Summary:**

Improvements in diet quality, with and without caloric restriction have been shown to improve CVD risk and to prevent the development of T2DM in individuals at risk; however, with regard to glycemic control in patients with T2DM, any dietary intervention resulting in significant weight loss may produce clinically meaningful benefits. Finally, dietary interventions with and without energy restriction that can improve cardiorespiratory fitness, even in absence of weight loss in patients with obesity, should be encouraged.

## Introduction

Type 2 diabetes mellitus (T2DM) and cardiovascular diseases (CVD) are leading causes of morbidity and mortality worldwide. In the United States (US) alone, approximately 11.3% and 5.5% of the adult population have T2DM [1] and CVD [2], respectively. Importantly, both T2DM and CVD disproportionally affect individuals of historically underrepresented communities [3], emphasizing the urgency to develop effective strategies that can be implemented across all individuals.

Lifestyle interventions represent the cornerstone therapy for T2DM and CVD, including both dietary modulation, physical activity (PA), and exercise training (ET). With regard to dietary interventions, several strategies have been proposed over the last few decades, resulting in the lack of universal approaches for the treatment of T2DM and CVD [4•]. In patients with overweight and obesity, weight loss achieved with dietary energy restriction remains first-line therapy not only to improve glycemic control in T2DM but also to improve cardiometabolic risk factors in patients with CVD. However, particularly with regard to improved CVD outcomes, the long-term benefits of weight loss have been rarely investigated with contrasting results. To the contrary, modulation of diet quality, with greater adherence to dietary patterns, even in the absence of energy restriction and weight loss, has been investigated in several large randomized controlled trials, clearly favoring improvements in diet quality over energy restriction in this setting [4•].

In this narrative review, we discuss the role of dietary interventions for the prevention and treatment of T2DM, with a focus on glycemic control and the treatment of CVD, in both primary and secondary prevention. Moreover, we discuss the importance of additional therapeutic strategies other than weight loss, such as improvements in cardiorespiratory fitness (CRF), in patients with obesity and other cardiometabolic risk factors.

## Weight Loss, Diet Quality, and PA/ET for the Prevention and Treatment of T2DM

Diet is a modifiable factor that plays an important role in both the prevention and management of T2DM [5, 6]. Dietary prescription is a cost-effective therapy that results in clinically meaningful improvements to glycemic control and can prevent the incidence of T2DM [7–10]. Benefits occur, in part, via reductions in body weight and increased diet quality [11], which will be addressed individually in the next paragraphs as well as in combination with PA/ET.

### Prevention of T2DM

Weight loss achieved by intensive lifestyle modification, i.e., energy restriction and increased PA, can prevent or delay the onset of T2DM in individuals at high risk [7, 9, 12–14]. Excess adipose tissue plays a key role in the development of T2DM. According to data from both the Multi-Ethnic Study of Atherosclerosis (MESA) and National Health and Nutrition Examination Survey (NHANES) groups, upwards of 53% of incident T2DM can be attributed to obesity alone [15]. Intentional weight loss achieved with lifestyle interventions is associated with significant improvements in glycemic control, micro- and macrovascular complications, as well as quality of life [16].

The Diabetes Prevention Program (DPP) demonstrated that in 3234 US adults with prediabetes and overweight or obesity, intensive lifestyle modification with a goal of 7% weight loss over 24 weeks resulted in 58% lower incidence of T2DM in the lifestyle intervention arm compared to the control arm over a mean 2.8 years follow-up [7]. In the Finnish Diabetes Prevention Study, which aimed for ≥ 5% weight loss over 4 years in 522 adults with impaired glucose tolerance (IGT) and overweight or obesity, a 15% (95% CI 7.2–23.2) absolute risk reduction for T2DM was achieved for the intervention group relative to the control group at year 6 [9]. The Da Qing Diabetes Prevention Study randomized 577 participants with IGT to diet, ET, diet with ET interventions, or control [8], but only included a weight loss component for participants with a body mass index (BMI) ≥ 25.0 kg/m^2^, for whom the goal was weight loss to a BMI of 23.0 kg/m^2^ [8]. Over 6 years, both diet and diet with ET were associated with a 31% and 42% reduced risk of developing T2DM, respectively [8].

Despite these impressive results, strategies for long-term maintenance are needed. After 15 years of follow-up with the DPP cohort, a shrinking difference in diabetes incidence was evident between placebo, metformin, and intensive lifestyle modification groups—with 62%, 56%, and 55% of the groups developing T2DM, respectively [17]. This was confirmed by a meta-analysis of T2DM prevention programs which found that while intensive lifestyle modification was associated with a sustained reduction in T2DM, effects were diminished over the follow-up period [18]. Long-term maintenance strategies must be tested for their impact on T2DM prevention post intensive lifestyle modification. Until then, it must be understood that short term intensive lifestyle intervention will lead to delayed, but not prevented cases of T2DM for many participants.

### Weight Loss in T2DM

Currently, the American Diabetes Association (ADA) recommends that individuals with T2DM and obesity utilize nutrition therapy to achieve and maintain a weight loss of at least 5% of baseline body weight [6]. Although some studies have noted that lower amounts of weight loss (2–5%) can result in decreases in HbA1c levels, most have shown nonsignificant effects. In comparison, weight-loss interventions reporting moderate weight loss (≥ 5%) have significant declines in HbA1c levels [19] and improvements in β-cell function [20]. However, higher magnitudes of weight loss (~15%) have been shown to result in the most beneficial effects [16]. The DiRECT trial observed that a 10 kg decrease in body weight over a 12-month period in 137 individuals with T2DM resulted in a 0.9% decline in HbA1c levels and T2DM remission in 46% of intervention participants [10]. Though this study was composed of a total dietary replacement for 3–5 months, it is important to note that PA was introduced afterwards and may have played a role in the reported 12-month data. Attrition in DiRECT was high, with 21% (*n*=32) participants withdrawing from the intervention group over the course of the study while none of the control participants discontinued participation [10]. At 24 months, T2DM remission had dropped to 35.6% of intervention participants—notably, weight regain was higher between 12 and 24 months in individuals who relapsed vs. those who maintained remission (7.09 vs. 4.25 kg respectively), highlighting the need to investigate long term maintenance strategies. The CALERIE study enrolled individuals without obesity or T2DM. This clinical trial randomized healthy and overweight individuals to either a 25% calorie restriction diet or ad-libitum control diet for 2 years. Kraus et al. [21] reported that individuals in the caloric restriction group maintained a 10% weight loss and increased in insulin sensitivity, highlighting the effectiveness of caloric restriction for long-term weight loss and glycemic improvements.

### Diet Quality Improvements in T2DM

Evidence demonstrates the importance of weight loss for the management and reduction of T2DM-related complications; however, research in recent years has highlighted the importance of individual nutrients and dietary patterns. The ADA has placed a focus on promoting healthy eating patterns, and current nutrition therapy recommends that individuals with T2DM focus on nutrient-dense, high-quality foods (Table 1) [22]. In fact, some data suggest that the quality of nutrients may be more important than the quantity [23] as poor diet quality [healthy eating index (HEI) < 65%] is often associated with poor glycemic control [24, 25]. However, not all studies have observed this relationship [26], as data from the Atherosclerosis Risk in Communities (ARIC) Study found that higher diet quality was strongly associated with lower CVD but not diabetes risk in 10,808 adults [27].

**Table 1:** Nutritional therapy for glycemic control in type 2 diabetes

	Recommendations
*General*	First-line treatment
	No superior macronutrient composition
*Weight loss*	BMI ≥ 25 kg/m^2^: achieve and maintain ≥ 5% weight loss
	BMI ≥ 30 kg/m^2^: achieve and maintain ≥ 15% weight loss
	Aim for 500–750 kcal/day energy deficit
*Diet quality*	Emphasis on high-quality nutrient-dense food
	Individualized based on personal needs and preferences
	< 2300 mg/day sodium
	Moderate alcohol intake
	Hydrate with water or no- to low-calorie sweetened beverages
*Potential dietary plans*	Ketogenic diet (5–10% carbohydrate intake of total energy)
	Low-carbohydrate diet (< 26% carbohydrate intake of total energy)
	Mediterranean diet

*BMI* body mass index

Although the optimal dietary pattern for T2DM remains unclear, clinical trials have noted several dietary programs that improve glycemic control and reduce complications. A recent network meta-analysis that compared 10 dietary approaches via a clustered ranking plot reported the ketogenic diet, defined as 5–10% carbohydrate intake of total energy intake, to be superior in regard to overall glycemic control compared to control (HbA1c: −0.73% [95% CI: −1.19, −0.28], fasting glucose: −0.53 mmol/L [95% CI: −2.86, 1.79]) [28]. Secondary analyses from the DIETFITS randomized clinical trial found that individuals who followed a ketogenic diet (*n* = 18) (carbohydrates minus fiber < 30 g/day) lost two times as much weight (10 vs. 5 kg) and had greater insulin resistance improvements (30 vs. 15%) compared to the overall DIETFITS population [29]. Although energy intake did not differ from the overall cohort, the authors do propose that “anchoring” occurred for those participants who were able to achieve a very low carbohydrate intake—meaning greater adherence to the dietary intervention and energy restriction may have in fact occurred even if this was not evident on statistical analysis [29]. The ketogenic diet is also not without side effects. In fact, it causes increases in low-density lipoprotein cholesterol (LDL-C), and whether it can be sustained for long periods of time is unclear as most studies are short in duration. Other research that focused on reducing overall carbohydrate intake has shown similar glycemic benefits. The ADA recommends that initial nutrition management should include the reduction of carbohydrates [16]. Systematic reviews have reported large clinical improvements in weight loss, insulin sensitivity, and diabetes remission (defined as HbA1c < 6.5%) within the first year of low-carbohydrate diets. However, these statistical differences compared to high-carbohydrate diets are often diminished after 12 months [30, 31].

The Mediterranean diet, a dietary plan rich in unsaturated fatty acids (UFA), has also shown strong potential for reducing fasting glucose levels [32] and subsequently T2DM risk [28, 33]. In a trial conducted by Esposito and colleagues [34], individuals with newly diagnosed T2DM (*n* = 215) were randomized to either a Mediterranean-style diet or low-fat diet. After 4 years, the Mediterranean diet resulted in greater weight loss and better glycemic control compared to the low-fat diet group. In fact, only 44% of individuals on the Mediterranean diet compared to 70% of individuals on the low-fat diet required antihyperglycemic pharmacotherapy. A characteristic of the Mediterranean diet that has been associated with these glycemic benefits is its high fiber content, which is usually two-fold higher than usual Western diets [35]. Across 15 studies in adults with T2DM, increasing dietary fiber by at least 10 g/day from baseline intake for 8 weeks was associated with a significant decrease in HbA1c levels [36].

### Combination Therapy: Diet, PA, and ET

It is often recommended that individuals with T2DM undertake lifestyle modifications, such as ET and nutrition therapy, simultaneously to achieve maximal benefits. The Look Action for Health in Diabetes (AHEAD) trial enrolled 5145 participants with T2DM and overweight or obesity and randomized them to an intensive lifestyle intervention (ILI) or diabetes support and education (DSE), i.e., control group [37]. Individuals were assigned the goal of losing 10% body weight through caloric deficit diets and 175 min/week of moderate-to-vigorous PA [38]. On average, individuals assigned to the intervention lost 8.6% of body weight in the first year, which was significantly correlated with improved glycemic control and robust reductions in HbA1C levels (7.3 to 6.6%). Out of the 5145 participants randomized, only 3% (*n* = 89) and 4% (*n* = 99) withdrew from ILI or DSE, respectively. Over the median follow-up of 9.6 years, ILI participants did partially regain but maintained a weight loss of 6% from baseline while DSE participants lost 3.5% from their baseline weight. In accordance with this modest difference in weight loss, differences in T2DM remission were significant, but not large between groups (ILI, 3.5% vs. DSE, 0.5% sustained remission for 4 years) [39].

Taken together, the current evidence suggests that both weight loss and diet quality play important roles in glycemic control and management of T2DM. Currently, the ADA recommends that individuals with T2DM and obesity focus on losing and maintaining at least 5% of baseline body weight and consuming nutrient dense and high-quality foods. It is important to note that there is no ideal macronutrient plan, and a variety of eating patterns can be considered as nutrition therapy requires individualization to optimize glycemic control and overall cardiometabolic health.

## Weight Loss, Diet Quality, and CVD

### Energy Restriction-Induced Weight Loss

**Fig. 1:**
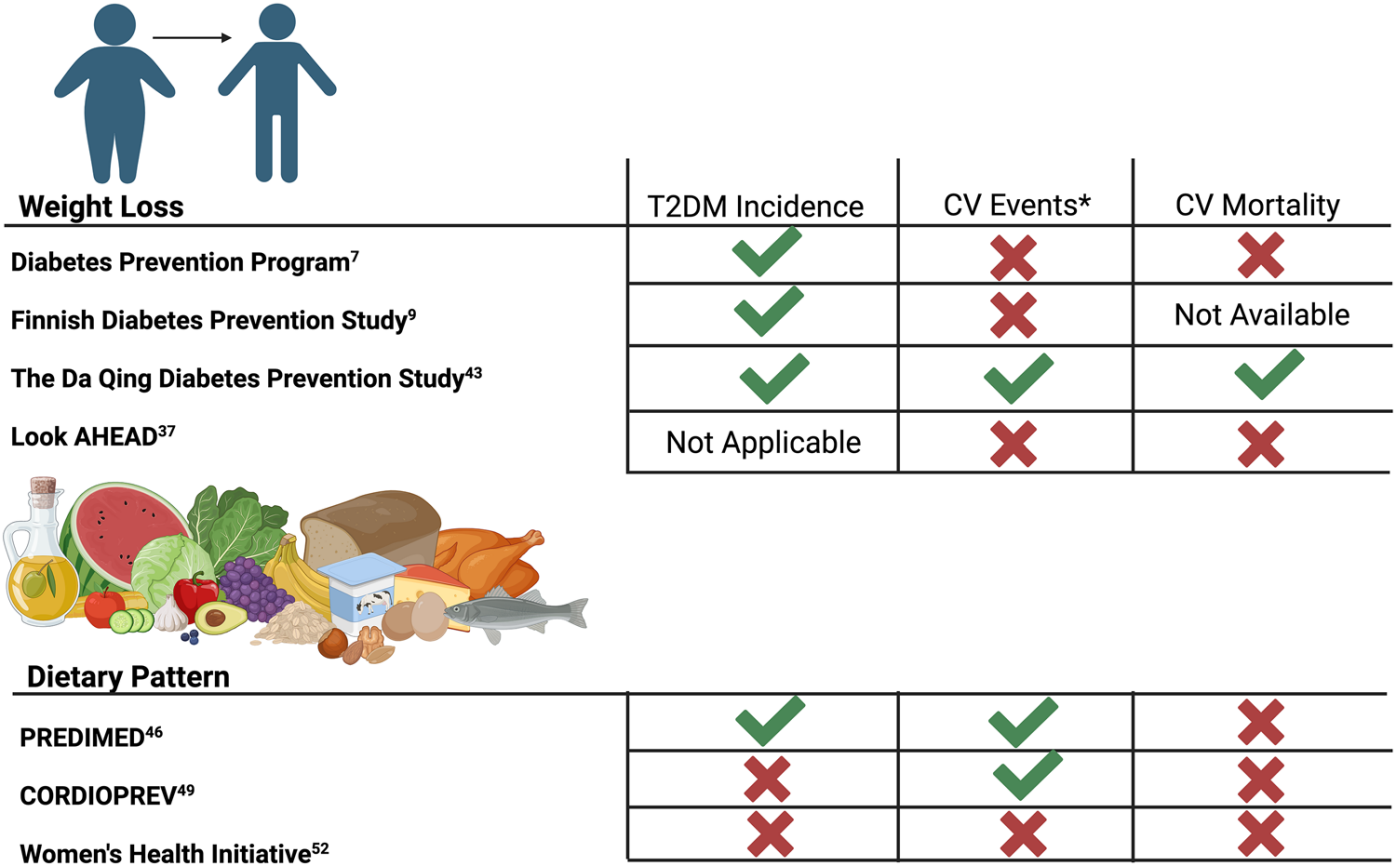
Energy restriction-induced weight loss and improvements in diet quality to prevent and treat type 2 diabetes mellitus and cardiovascular diseases. Created with BioRender.com. Abbreviations: CV, cardiovascular; T2DM, type 2 diabetes mellitus

The success of weight loss achieved by intensive lifestyle modification in preventing or delaying T2DM has largely not translated to CVD outcomes (Fig. 1). After 15 years of follow-up, DPP’s intervention group still demonstrated a 27% lower incidence of T2DM relative to the control group (HR: 0.73 [95% CI 0.65–0.83]) [17], but at 21 years, no between-group differences were observed in major CVD events (HR: 1.14 [95% CI 0.87–1.50]) nor mortality (HR: 1.18 [95% CI 0.77–1.81]) [40, 41]. Similarly, after 10 years of follow-up in the Finnish Diabetes Prevention Study, the intervention group did not display reduced CVD events (HR:1.04 [95% CI: 0.72–1.5]) relative to control [42].

In adults with established T2DM, intentional weight loss has also failed to translate to CVD protection. In the Look AHEAD trial, despite maintenance of greater weight loss in intervention vs. control groups (6.0% vs. 3.5%), after a median follow-up of 9.6 years, no between-group differences were observed in the primary outcome of major CVD events (HR intervention: 0.95 [95% CI 0.83–1.09]) [37].

The Da Qing Study presents an exception to other intensive lifestyle modification trials discussed. After 30 years of follow-up, a delayed onset (3.96 years [95% CI 1.25–6.67]) and lower incidence of T2DM (HR 0.61 [95% CI 0.45–0.83]) was noted in the combined intervention groups along with lower CVD events (HR 0.74, 95% [CI 0.59–0.92]) and mortality (HR 0.67, [95% CI 0.48–0.94]) relative to control participants [43]. There are numerous potential reasons for this difference, including a relatively long follow-up period. Notably, at 20 years of follow-up, no between group differences had emerged for CVD events nor mortality [44]. Additionally, 40% of participants in Da Qing presented with a BMI < 25.0 kg/m^2^, and all participants assigned to a diet group, regardless of BMI, were counseled to improve dietary quality by reducing alcohol and simple sugar consumption and increasing vegetable intake [8]. This brings up an important question—what evidence exists that changing dietary quality, in the absence of significant weight loss, can improve CVD outcomes?

### Diet Quality Improvements to Prevent and Treat CVD

The landmark Prevención con Dieta Mediterránea (PREDIMED) trial randomized 7447 adults at high cardiovascular risk 1:1:1 to a Mediterranean diet (MedDiet) supplemented with either 1 L per week per household of extra virgin olive oil (EVOO) (the goal was to consume at least 4 tablespoons per day) or 30 g daily of mixed nuts and seeds or a low-fat diet, which was considered the control group [45]. After a median follow-up period of 4.8 years, the MedDiet with EVOO and mixed nuts resulted in 30% relative risk reduction of the primary endpoint of acute myocardial infarction, stroke, or CVD mortality (HR EVOO: 0.70 [95% CI 0.53–0.91] and HR nuts: 0.70 [95% CI 0.53–0.94]). During this period, attrition rates were 11.3% in the low-fat control and 4.9% in the MedDiet groups. In 2018, PREDIMED was retracted and republished due to randomization deviations at 2 sites, removing these sites from analyses did not alter outcomes [46]. Adherence in the MedDiet groups was confirmed via objective biomarkers—urine hydroxytyrosol in those assigned to EVOO and plasma alpha-linoleic acid in those assigned to mixed nuts—both biomarkers increased from baseline only within their respective group, suggesting adherence to the intervention. While the low fat group did not have a specific biomarker of adherence nor a specific intake target, they self-reported decreasing total fat intake by only 2% of total kilocalories (kcals) (39.0 to 37.0%). Additionally, in 418 PREDIMED participants without baseline T2DM, assignment to the MedDiet reduced incidence of new-onset T2DM by 52% over a median of 4 years follow-up compared with the low-fat control group (HR EVOO: 0.49 [95% CI 0.25–0.97] and HR nuts: 0.48 [95% CI 0.24–0.96]) [47, 48].

Recently, the Coronary Diet Intervention with Olive Oil and Cardiovascular Prevention (CORDIOPREV) study enrolled 1002 patients with established coronary heart disease and randomized them to MedDiet, with 1 L EVOO allocated per week per household, or low fat control. After 7 years follow-up, the MedDiet reduced the primary endpoint of major CVD events by 26% compared to the low-fat diet control (HR 0.73 [95% CI 0.55–0.97]) [49••]. In 462 CORDIOPREV participants without baseline T2DM, no between-group difference in T2DM incidence was observed over 60 months of follow-up. As in PREDIMED, attrition was greater in the low-fat control, 17% (*n* = 86/500), compared to the MedDiet group, 9% (*n* = 46/500). Adherence was not measured by objective biomarkers of intake but by dietary screeners—the MedDiet group reported increasing their MedDiet score (range 0–14) by 2 points while the low-fat group reported a reduction in total fat intake from 36 to 32% of daily kcals, not quite meeting the target of < 30% kcals from fat. A subgroup analysis suggested a low-fat diet may be more effective in preventing T2DM in individuals with established prediabetes, although results differed by which prediabetes diagnostic criteria was utilized [50].

Most major clinical trials have failed to demonstrate that weight loss, achieved through intensive lifestyle modification, results in improved CVD outcomes in adults with prediabetes or T2DM, regardless of T2DM delay or prevention [37, 40–42]. Few large randomized controlled trials have examined the impact of dietary quality interventions on CVD outcomes. A high-unsaturated fatty acids MedDiet is more effective in primary (PREDIMED) and secondary (CORDIOPREV) prevention of major CVD events than a low-fat diet [45, 46, 49••]. However, the impact of the MedDiet on T2DM prevention remains unclear, and appropriately powered randomized controlled trials are urgently needed to assess the impact of this diet on T2DM incidence [47, 48, 50, 51]. Moreover, both PREDIMED and CORDIOPREV have been conducted in Spain, in a population with a relatively higher adherence to a MedDiet at baseline. Whether these results can be replicated in the US is unknown. Notably, the Women’s Health Initiative randomized controlled dietary modification trial conducted in the US did not find any benefits on CVD in post-menopausal women randomized to a low-fat diet or control group [52].

Adherence to other healthy dietary patterns, i.e., plant-based and Dietary Approaches to Stop Hypertension (DASH), has frequently demonstrated associations with reduced incidence of CVD and T2DM, but major randomized controlled trials are necessary to identify the impact of these dietary patterns on T2DM incidence and CVD outcomes [53, 54].

### New Therapeutic Targets: Focus on CRF

CRF provides an integrated measure of the pulmonary, cardiovascular, and skeletal muscle systems and is an independent predictor of mortality [55]. In 2316 men with T2DM followed for an average of 15.9 years, participants were categorized by BMI and CRF level (low, moderate, or high) [56••]. An inverse gradient was observed for CVD death across CRF categories within each BMI category. Notably, individuals with obesity and moderate or high CRF exhibited half the CVD mortality risk compared to individuals with normal weight and low CRF [56••]. In a meta-analysis that included 24 studies and 84,323 participants, for every one metabolic equivalent of task (MET) increase in CRF, there was a 15% lower risk of CVD events [55]. Moreover, two meta-analyses reported a 5–8% lower risk of T2DM for every one-unit MET increase in CRF [57, 58]. These findings demonstrate the importance of CRF independent of body weight and the utility of CRF as a clinical marker of disease incidence and mortality [56••, 59].

CRF as defined by the Fick equation is equal to cardiac output multiplied by the difference in arterial and venous oxygen concentrations. Thus, interventions aiming to improve CRF may target these factors contributing to CRF. Dietary interventions have the potential to improve CRF through several mechanisms, including central (e.g., cardiac function) and peripheral factors (e.g., skeletal muscle quality, oxygen transport in the blood). For instance, initial findings in cross-sectional studies have reported that higher dietary quality, as assessed by MedDiet scores and the alternate HEI, was associated with higher CRF [60, 61]. Dietary interventions examining the impact of improved dietary quality and/or energy restriction on CRF and its determinants have also been conducted in populations with CVD and T2DM.

In one of the largest investigations of the impact of dietary intervention on CRF to date, patients with T2DM in a subset of the Look AHEAD trial (3942 participants) underwent graded exercise treadmill testing to assess CRF. After 4 years, the ILI group showed a 5.4% improvement in CRF relative to − 0.83% decline in the DSE group, adjusting for baseline CRF (*p*<0.0001) [62]. Increases in CRF were also associated with improvements in glycemic control. Importantly, individuals not taking a diabetes medication or insulin and those without prior CVD or metabolic syndrome had significantly greater improvements in CRF [62]. A smaller (*n*=53) 1-year intervention of counseling to induce moderate weight loss (500 kcal daily energy restriction), improve dietary quality and increase PA in men who underwent coronary artery bypass grafting resulted in a 13% increase in CRF and improved diastolic function [63]. In this study, the presence of T2DM was a significant predictor of change in diastolic function [63].

Outside of weight loss interventions, changes in dietary quality alone or paired with ET may have the potential to improve CRF, although trials have been small (*n* < 100). In patients with heart failure (HF), A 3-month DASH diet intervention (*n* = 24) resulted in improved 6-min walk distance (i.e., submaximal exercise capacity) relative to a control group following HF recommendations (292 vs. 197 m, *p* = 0.018) [64]. In a pilot study of 13 hypertensive patients with HF with preserved ejection fraction (HFpEF), consuming a DASH/sodium-restricted diet for 21 days led to improvements in blood pressure measured in clinic, 24-h ambulatory blood pressure, and carotid-femoral pulse wave velocity as well as reduced urinary F2-isoprostane levels [65]. Participants also demonstrated improved submaximal exercise capacity over the study period via 6-min walk test distance (313 ± 86 to 337 ± 91 m; *p* = 0.006) [65]. In another publication from the same study, participants exhibited improved stroke volume, ejection fraction, and systemic vascular resistance [66]. Another 12-week single arm pilot study of 9 individuals with HFpEF receiving dietary advice to increase consumption of foods rich in unsaturated fatty acids reported a trend towards an increase (*p* = 0.069) in CRF [67]. Finally, in another study of individuals enrolled following a myocardial infarction, Mediterranean diet recommendations combined with low-volume or high-volume high intensity aerobic interval training resulted in 15% and 22% improvements in CRF, respectively; however, dietary adherence/change was not reported [68]

## Conclusions

Weight loss represents an effective therapy for the prevention and treatment of T2DM, particularly with regard to improvements in glycemic control. However, weight loss alone has not been associated with the prevention of CVD in this population in most studies. To the contrary, improvements in diet quality, particularly by promoting a dietary pattern rich in unsaturated fatty acids (i.e., MedDiet), have shown to prevent CVD in both primary and secondary prevention, even in the absence of weight loss (Fig. 1). Ongoing clinical trials will answer the question on whether weight loss in addition to MedDiet can further reduce the risk for CVD. Moreover, improvements in CRF and its determinants have been recently identified as an alternative therapeutic strategy to weight loss, also in the setting of obesity. Prospective clinical trials comparing whether CRF or weight loss is a stronger predictor for CVD are urgently needed to guide clinicians on the most effective therapeutic strategy to extend the lifespan of individuals. Moreover, large randomized controlled clinical trials investigating the effects of dietary interventions on CVD are urgently needed in the US.

## Data Availability

No datasets were generated or analysed during the current study.
